# Supplementary material for: Content-rich biological network constructed by mining PubMed abstracts
Source: BMC Bioinformatics. 2004 Oct 8;5:147. doi: 10.1186/1471-2105-5-147 (PMC528731; doi:10.1186/1471-2105-5-147)
Supplement: Additional File 2 — The original results of the above study (non-essential files are deleted to keep the file size under the limit set by BMC bioinformatics). [file 1471-2105-5-147-S2.bz2 › chilibotAdditionalFile2/dip05/465ID9660793E10492/html/CXCR3_CXCL10.html]

 


 **CXCR3** and **CXCL10** 
  
Found 773 abstracts in PubMed, retrieved 05.  
 

 What does Google say? 
 PDF only 
| .edu only 

---

**Interactive relationship** (e.g. stimulation, inhibition, etc)

**Neutral relationship**- The chemokine receptor  **CXCR3**  is predominantly expressed on T lymphocytes, and its agonists CXCL9,  **CXCL10**  and CXCL11 are IFN gamma inducible chemokines that promote Th1 responses.  Ref: 12884299 Eur J Immunol, 2003

**Non-interactive relationship** (e.g. studied together, co-existance, homology, etc.)

- Correspondingly, the rate or intensity of inflammation in IL 10 mice treated with anti IP 10  [ **CXCL10** ]   [ **CXCR3** ]  Abs showed improved scoring of inflammation, compared with control IL 10 mice.  Ref: 12874231 J Immunol, 2003
- This study provides important and novel information regarding IP 10  [ **CXCL10** ]   [ **CXCR3** ]  as a target for the treatment of colitis.  Ref: 12874231 J Immunol, 2003
- Specifically, IP 10  [ **CXCL10** ]   [ **CXCR3** ]  inhibition in IL 10 mice attenuates the associated increases in serum and or local amyloid A, IL 2, IL 6, TNF alpha, IFN gamma, IL 1alpha, and IL 1beta with colitis as compared with IL 10 mice that develop colitis similar to human Crohn s disease.  Ref: 12874231 J Immunol, 2003
- High levels of RANTES CCL5, MCP 1 CCL2, IP 10  [ **CXCL10** ]   [ **CXCR3** ]   **CXCL10** , and Mig CXCL9 simultaneously with mononuclear cell infiltration in the lungs was found.  Ref: 12875978 Am J Pathol, 2003
- CCR3 functional responses are regulated by both  **CXCR3**  and its ligands CXCL9,  **CXCL10**  and CXCL11.  Ref: 12884299 Eur J Immunol, 2003
- Consistent with these results, infected cells from WT mice preferentially migrated in response to IP 10  [ **CXCL10** ]   [ **CXCR3** ]   **CXCR3**  ligand, while those from GKO mice migrated in response to eotaxin CCL11 CCR3 ligand.  Ref: 12875978 Am J Pathol, 2003
- IFN gamma inducible protein 10 IP 10  [ **CXCL10** ]   [ **CXCR3** ]  and  **CXCR3**  are highly expressed at sites of colitis.  Ref: 12874231 J Immunol, 2003
- 2 Interferon IFN inducible anti viral proteins, including IFIT1, OAS1 and Mx1, and Th1 chemoattractant IP 10  [ **CXCL10** ]   [ **CXCR3** ] , were up regulated predominantly 6 to 24 hours after stimulation.  Ref: 12887736 BMC ImmunolBMC Immunol, 2003
- Our findings show that IP 10  [ **CXCL10** ]   [ **CXCR3** ]  significantly contributes to the development of Th1 and inflammatory responses.  Ref: 12874231 J Immunol, 2003
